# The Cost of Simplifying Air Travel When Modeling Disease Spread

**DOI:** 10.1371/journal.pone.0004403

**Published:** 2009-02-06

**Authors:** Justin Lessler, James H. Kaufman, Daniel A. Ford, Judith V. Douglas

**Affiliations:** 1 Bloomberg School of Public Health, Johns Hopkins University, Baltimore, Maryland, United States of America; 2 Health Informatics Research, IBM Almaden Research Center, San Jose, California, United States of America; University of Oxford, United Kingdom

## Abstract

**Background:**

Air travel plays a key role in the spread of many pathogens. Modeling the long distance spread of infectious disease in these cases requires an air travel model. Highly detailed air transportation models can be over determined and computationally problematic. We compared the predictions of a simplified air transport model with those of a model of all routes and assessed the impact of differences on models of infectious disease.

**Methodology/Principal Findings:**

Using U.S. ticket data from 2007, we compared a simplified “pipe” model, in which individuals flow in and out of the air transport system based on the number of arrivals and departures from a given airport, to a fully saturated model where all routes are modeled individually. We also compared the pipe model to a “gravity” model where the probability of travel is scaled by physical distance; the gravity model did not differ significantly from the pipe model. The pipe model roughly approximated actual air travel, but tended to overestimate the number of trips between small airports and underestimate travel between major east and west coast airports. For most routes, the maximum number of false (or missed) introductions of disease is small (<1 per day) but for a few routes this rate is greatly underestimated by the pipe model.

**Conclusions/Significance:**

If our interest is in large scale regional and national effects of disease, the simplified pipe model may be adequate. If we are interested in specific effects of interventions on particular air routes or the time for the disease to reach a particular location, a more complex point-to-point model will be more accurate. For many problems a hybrid model that independently models some frequently traveled routes may be the best choice. Regardless of the model used, the effect of simplifications and sensitivity to errors in parameter estimation should be analyzed.

## Introduction

Air travel plays an important role in facilitating the spread of many infectious diseases, and systems that model that spread may need to take it into account. Crépey and Barthélemy [1] analyzed 30 years of data on seasonal influenza outbreaks in United States and 20 years of data for France, concluding that, in the United States, “realistic modeling of the spread of epidemics at the interstate level may only need to take air transportation into account,” whereas modeling France would require several transportation modes. Brownstein et al. [2] analyzed the spread of influenza in the United States for 9 years (1996–2005) using signal processing methods, and found that domestic air travel volume predicted the rate of influenza spread, and international air travel affected the timing of influenza mortality. In their view, the “delayed and prolonged influenza season” that followed the ban on air travel in the United States after the September 11, 2001 terrorist attacks provided empirical evidence for the role of air travel in long range disease spread. Subsequent analysis in defense of their findings included data from a 30-year period [3]. While air travel is clearly important for the long range spread of many infectious diseases, as illustrated by the SARS epidemic [4], regionally its importance may be diminished. Viboud et al. [5] found that regional spread correlated most closely with the movement of people to and from their workplaces, and that the “magnitude of impact” of air travel remained unclear in comparison.

In modeling air travel, as with many aspects of disease spread, the temptation is to include all possible detail, but this may lead to unwieldy, complex systems that are difficult to validate and slow to run. When stochastic models are used, this computational complexity can seriously impact our ability to run the tens of thousands of simulations may be necessary for valid results.

Models of air travel (or travel in general) may be integrated into epidemiological models in different ways, but at some level must account for the movement of, or contact between, people at distant locations. The details of this integration and of the models themselves are not our focus here, but an example is the open source Spatial Temporal Epidemiological Modeling (STEM) project [6]–[8], in support of which we performed this analysis. The modeler's problem is to come up with models of transportation that capture the contacts and movement important to disease spread, yet are simple enough to be computationally efficient and fit to (often minimal) data. Air travel introduces long range and high degree connectivity to any transportation network that can be computationally expensive. As such it is important to consider the accuracies and inaccuracies, as well as the computational cost of alternative air transportation models.

The appropriate level of abstraction, and indeed the importance of air travel itself, is dependent on the disease being studied and the question being asked. The analysis presented here focuses on diseases spread by person-to-person contact, which includes many of those where rapid control might be required, e.g., influenza, smallpox [9], [10]. We focus primarily on a single metric of the effect of air travel: the frequency of long range introductions. However, transmission during air travel, whether on the plane or at the airport, may also be important [4].

As part of our work developing STEM, we evaluated a simplified air transportation model, where all individuals flow through a single hub, in comparison with a fully saturated model where all routes are modeled individually, and a “gravity” model where the probability of travel between airports is scaled by their physical distance.

In this article we attempt to characterize the errors associated with the simplified model in a manner meaningful to the disease modeler. The level of complexity required for a model largely depends on the question being asked; by specifying the type and magnitude of errors, we hope to aid disease modelers in deciding if using a simplified air transport model will substantively impact their conclusions.

## Methods

We obtained data on individual tickets within the United States for all of 2007 from the U. S. Department of Transportation Research and Innovative Technology Administration Bureau of Transportation Statistics (RITA-BTS). Tickets give the origin and destination of full trips, rather than individual flights. The RITA-BTS ticket data (DB1BTicket from the Airline Origin and Destination Survey) are a sample of 10% of U.S. tickets from reporting carriers. Using this model we calculated the probability of a trip originating at any airport *A*, terminating at any other airport *B*, as 
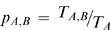
 where *T_A,B_* is the number of trips from *A* to *B*, and *T_A_* is the total number of trips originating at *A*. This defines the saturated, point-to-point model.

In order to account for the possibility of flights on unseen routes, and ensure comparability between models, we assigned 0.1 trip per year on every possible route not seen in the RITA-BTS data. These unseen trips account for 0.01% of the trips considered in this analysis.

The simplified model we used is a “pipe” model, in which individuals flow in and out of the air transport system based on the number of arrivals and departures from a given airport (i.e., there is no explicit modeling of individual routes). In this model, the flow of passengers in the air transportation network is considered to be like that of an incompressible fluid flowing through pipes where airports are sources and sinks of fluid. The more traffic through a given airport, the more fluid is flowing and the larger the associated “pipe” into the network. Since any traveler in the global transportation system has some probability of mixing with any other traveler (either on a flight or during a flight change at some hub), the pipes of all diameters join in some abstract hub in this model. Point-to-point travel is then determined by the product of the probability of travel from the origin, to the destination, normalized by the total travel. Under this model the probability of a trip from origin *A* terminating at *B* is the proportion of all trips at any location ending at *B*:
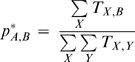
To determine whether differences between *p_A,B_* and p^*^_A,B_ could best be explained by the distance between the two locations, we considered a third “gravity” model of transport. Gravity models have proven useful in general (i.e., non-mode specific) models of transportation [11], and assume that the probability of an individual going from point *A* to point *B* is inversely proportional to some power of the distance between those locations. Under this model the probability that a trip from origin *A* terminates at *B* is:
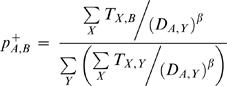
where *_DA,_B* is the distance between *A* and *B* calculated based on their latitude and longitude using the spherical law of cosines. We determined the appropriate *β* for this model by finding the value that maximized the likelihood of the data using a Newton type algorithm (as implemented in the nlm function in the *R* statistical language) [12]. Note that for a *β* of 0 this model reduces to the pipe model. More advanced gravity models have been developed wherein the probability of travel to/from a population center scales with population to some exponent. Including these exponents can increase the accuracy of the model [13].

In infectious disease modeling we are interested in the rate of introductions from *A* to *B*, *λ_A,B_*, and the overall rate of introductions into a given area, *θ_B_*. Differences in these can be characterized in terms of their ratio, or their absolute difference. The latter is of more interest for the infectious disease modeler, because it can be used to quantify the expected rate of false introductions (or missed introductions) over the course of the epidemic. Table 1 shows these relations. We do not calculate *θ_B_* over the course of the epidemic as this quantity does not have a closed form solution. All analysis was done using the *R* statistical package [12].

**Table 1:** Rate of introductions into a given airport.

Difference in rate of introductions from *A* to *B* at a particular point in the epidemic	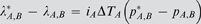
Difference in number of introductions from *A* to *B* over the course of the epidemic	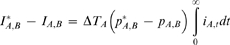
Difference in overall rate of introductions into *B*	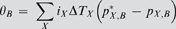

## Results

The maximum likelihood estimate of *β* for the gravity model was −0.0527. The probability of a trip from a given origin to a given destination is never more than 1.12 times more likely or less than 0.74 times less likely under the gravity model than the pipe model, and 95% are between 0.91 and 1.08 times as likely. Overall, the gravity model is not significantly different from the pipe model, and will not be considered further.

**Figure 1:**
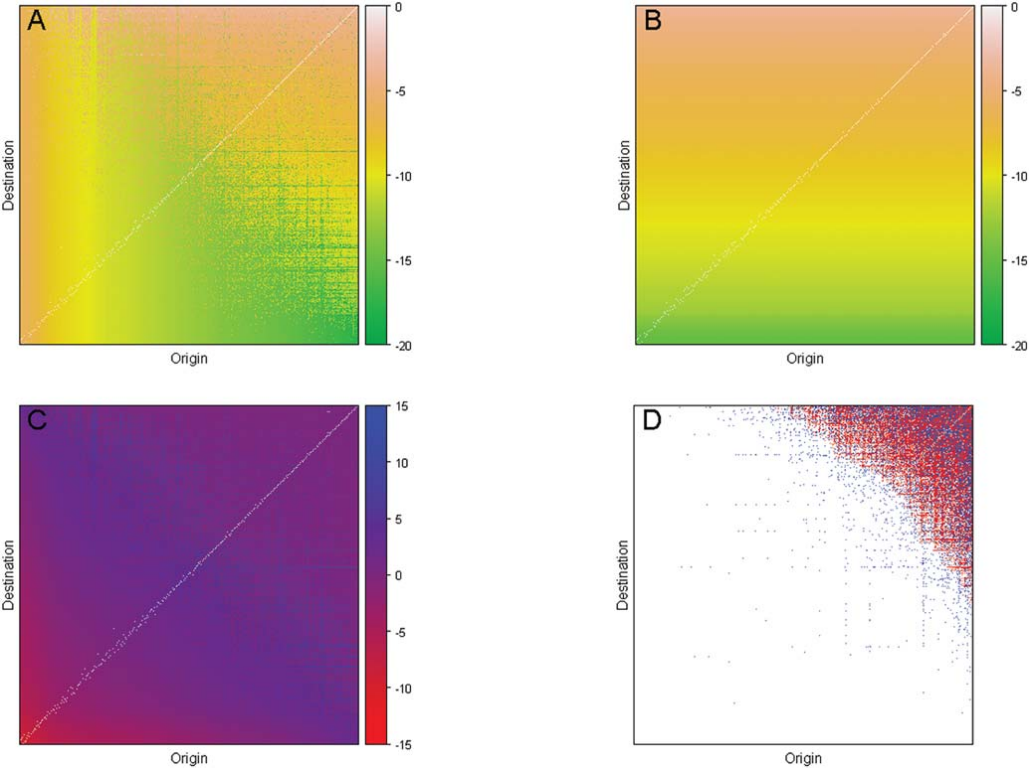
Comparison of the pipe and saturated models of air transport. Legend: In all four graphs origins are ordered left to right by increasing airport traffic, and destinations are ordered bottom to top by increasing airport traffic. (A) The log-probability a trip from a given origin airport is to a particular destination airport under the saturated model. (B) The log-probability a trip from a given origin airport is to a particular destination airport under the pipe model. (C) The log probability ratio of the pipe model versus the saturated model. (D) Trips for which the rate of disease introductions from a fully infected location is overestimated by at least one individual per day (red) or underestimated by one individual per day (blue) under the pipe model.

In Figure 1, panels A and B make clear the essential difference between the two models. In the pipe transport travelers are equally likely to go to a particular destination regardless of where the trip originated, whereas in the saturated model tickets from smaller airports are more likely to terminate at larger airports, and the destinations of tickets from larger airports are more evenly distributed across destinations. When comparing the relative probability that a person from a given airport will go to another one (panel C), we find that the frequency with which trips from busy airports end at other busy airports is nearly correct under the pipe model, but that the probability a trip from a busy airport is to a less busy airport is underestimated by the pipe model. For trips originating from smaller airports, the probability the destination is another small airport is overestimated by the pipe model.

Of interest to the infectious disease modeler is the frequency with which a disease will be introduced under the pipe model, and not introduced under the full model (and vice-versa). To quantify this, we looked at the difference in the rate of introductions from an origin to each particular destination under the two models under the assumption that everyone at the origin is infected with the disease. Using this metric, we found that in only 10% of routes will the rate of introductions be over- or underestimated by at least one person per day, and this over- or underestimation will tend to occur on the most traveled routes (Figure 1D). In 2% of routes, the difference in rates is at least 10, and in 0.05% of cases is it at least 100. Only for four routes (JFK→LAX, LAX→JFK, JFK→SFO, SFO→JFK) is the difference at least 500. All of these routes are frequently traveled (≥600 passengers a day) cross country routes where the pipe transport model significantly underestimates the probability of the trip (and hence the number of introductions).

**Figure 2:**
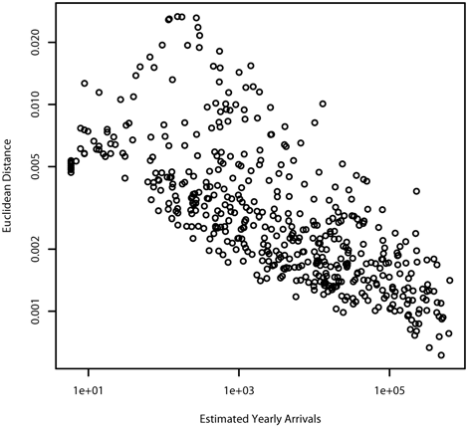
Comparison of origin for arriving passengers under the two models. Legend: The Euclidian distance between the vectors representing the probability that a particular arrival at an airport is from a particular origin under the pipe-transport and saturated models versus the estimated number of yearly arrivals at the airport. For airports with larger numbers of arrivals the pipe model more accurately approximates the true distribution of arrivals.

A final method of evaluating the extent to which the pipe model approximates the full model involves comparing the difference between the mixture of flight origins for individuals coming into a given airport under the saturated model and the pipe model. This can be characterized by the calculating the Euclidean distance between the vector of percentages of arrivals coming from each airport under the saturated model and the pipe model. Those airports with the fewest arrivals per year have a larger difference in the makeup of their arrivals between the predictions of the pipe model and the saturated model, as shown in Figure 2.

## Discussion

While the simplified pipe model of air travel provides a rough approximation of actual air travel, it has several shortcomings. Most of these can be traced back to the pipe model's overestimation of the number of small town to small town trips. The other simplified model considered, a gravity model which takes into account distance, has similar problems and offers little benefit for the increased complexity.

For those highly infectious disease where air transportation plays an important role, underestimation of the number of disease introductions that would occur from travel between major western and eastern populations centers (e.g., Los Angeles and New York) may result in models that underestimate the speed with which the a disease will cross the country. Similarly, the overestimation of the number of locations from which people travel to less busy airports may lead to models where diseases will more rapidly reach locations that might remain protected for a longer period of time. However, for most routes, the size of these effects are relatively small, and the former problem may be correctable by a hybrid model, where frequently traveled routes are treated independently (amplified). Computationally a pipe model offers an enormous advantage as it captures disease transmission by air travel with a 2N edged graph, compared with the point-to-point model that requires 2N^2^ edges.

In modeling the large scale regional and national effects of disease, the pipe model may be adequate, if the most important driver of disease spread is local contact and transportation modeling serves only to allow the disease to make long distance jumps across the country. If we are interested in the specific effects of interventions on particular air routes, or the time for the disease to reach a particular location, a more complex point-to-point model will be more accurate. For the most sophisticated and realistic simulations even a model of point-to-point trips may be too much of a simplification, as contact within airports during transit may play an important role in transmission. There may be other factors that lead the investigator to choose one model over the other, for instance, in the pipe model it is straight forward to implement mixing within the air transport system, whereas this may be more difficult in a point to point model.

Regardless of which model is used, it is important that the implications of any simplifications, or errors in parameter estimation (which become more likely as model complexity increases), are analyzed so that the appropriate level of complexity for the problem at hand may be selected.

## References

[1] Crépey P, Barthélemy M (2007). Detecting robust patterns in the spread of epidemics: a case study of influenza in the United States and France.. American Journal of Epidemiology.

[2] Brownstein JS, Wolfe CJ, Mandl KD (2006). Empirical evidence for the effect of airline travel on inter-regional influenza spread in the United States.. PLoS Medicine.

[3] Brownstein M, Wolfe CJ, Mandle KD (2006). Air travel and the spread of influenza: authors' reply.. PLoS Medicine.

[4] Mangili A, Gendreau M (2005). Transmission of infectious disease during commercial air travel.. The Lancet.

[5] Viboud C, Miller MA, Grenfell BT, Bjørnstad ON, Simonsen L (2006). Correspondence. Air travel and the spread of influenza: important caveats.. PLoS Medicine.

[6] Eclipse Foundation (2008). Open Health Framework Spatial Temporal Epidemiological Modeling project.

[7] Ford DA, Kaufman JH, Eiron I (2006). An extensible spatial and temporal epidemiological modeling system.. International Journal of Health Geographics.

[8] Kaufman JH, Conant JL, Ford DA, Kirihata W, Jones B, Zeng D Assessing the accuracy of spatiotemporal epidemiological models.

[9] Morens DM, Folkers GK, Fauci AS (2004). The challenge of emerging and re-emerging infectious diseases.. Nature.

[10] Morse SS (1995). Factors in the emergence of infectious diseases.. Emerging Infectious Disease.

[11] Viboud C, Bjørnstad OT, Smith DL, Simonsen L, Miller MA (2006). Synchrony, waves, and spatial hierarchies in the spread of influenza.. Science.

[12] R Development Core Team (2008). R: A language and environment for statistical computing. Vienna, Austria.. http://www.R-project.org.

[13] Rodrigue JP, Comtois C, Slack B (2006). The geography of transport systems.

